# Baclofen acts in the central amygdala to reduce synaptic transmission and impair context fear conditioning

**DOI:** 10.1038/s41598-018-28321-0

**Published:** 2018-07-02

**Authors:** A. J. Delaney, J. W. Crane, N. M. Holmes, J. Fam, R. F. Westbrook

**Affiliations:** 1 School of Biomedical Sciences, Charles Sturt University, Orange, NSW 2800 Australia; 2 School of Medicine, University of Tasmania, Hobart, TAS 7000 Australia; 3 School of Psychology, University of New South Wales, Sydney, NSW 2052 Australia

## Abstract

The two main sub-divisions of the Central amygdala (CeA), the lateral-capsular (CeA-LC) and the medial (CeA-M), contain extensive networks of inhibitory interneurons. We have previously shown that activation of GABA_B_-receptors reduces excitatory transmission between axons of the pontine parabrachial nucleus and neurons of the CeA-LC by inhibiting glutamate release from presynaptic terminals^13^. Here we have characterised GABA_B_-receptor activation on other excitatory and inhibitory projections within the CeA. Using whole-cell, patch-clamp recordings, we found that the GABA_B_-receptor agonist baclofen significantly reduced excitatory and inhibitory transmission from all tested inputs into the CeA-LC and CeA-M. In all but one of the inputs, reductions in transmission were accompanied by an increase in paired pulse ratio, indicating that presynaptic GABA_B_-receptors acted to reduce the release probability of synaptic vesicles. To examine the impact of GABA_B_-receptors in the CeA on contextual fear-conditioning, we infused baclofen into the CeA immediately prior to training. Compared to vehicle-infused rats, baclofen-infused rats displayed significantly less freezing both during the final stages of the training period and at test 24 hours later. The results of this study demonstrate that, by suppressing excitatory and inhibitory transmission, activation of presynaptic GABA_B_-receptors in the CeA inhibits the development of context conditioned fear.

## Introduction

During contextual fear-conditioning an association is formed between the neutral, sensory stimuli present in the context (the conditioned stimuli) and a noxious, sensory stimulus, such as a foot-shock (the unconditioned stimulus), delivered while the animal is in the context. Following pairing of the context and the unconditioned stimulus, future exposure to the context elicits a suite of autonomic, endocrine and behavioural responses (the conditioned response)^1^. The formation of these fear memories occurs within the amygdala, an almond shaped structure deep within the medial temporal lobe. Within this structure, three sub-regions are critical for the acquisition and expression of fear-memories - the lateral amygdala and the basal amygdala (together, the basolateral amygdala), and the central amygdala (CeA)^1^. Early models of fear-conditioning proposed a division of duties between these three amygdala sub-regions, with the basolateral amygdala critical for the formation of fear memories and the CeA responsible for initiating fear-memory responses^2^. However, it is now apparent that the CeA is also involved in the acquisition of fear-memories^3^.

The CeA is composed of a lateral-capsular division (CeA-LC) and a medial division (CeA-M). Neurons within the CeA-M project to a number of extra-amygdala brain regions and are responsible for co-ordinating the autonomic and behavioural components of the fear responses^3^. At least two populations of inhibitory interneurons are present in the CeA-LC: CeA-LC_on_ neurons (protein kinase C-δ negative and somatostatin-positive), that increase their activity in response to conditioned stimuli, and CeA-LC_off_ neurons (protein kinase C-δ positive and somatostatin-negative), that decrease their activity in response to conditioned stimuli^4^. The CeA-LC_off_ neurons inhibit output neurons of the CeA-M, but the CeA-LC_off_ neurons are inhibited by the CeA-LC_on_ neurons^5^. Thus, activation of CeA-LC_on_ neurons results in disinhibition of output neurons of the CeA-M neurons, permitting the generation of conditioned responses^4,5^. However, neurons within the CeA-LC are also involved in the acquisition and consolidation of fear-memories^3^. Fear conditioning potentiates the excitatory connection onto somatostatin-positive (CeA-LC_on_) neurons, and this is required for the acquisition of conditioned fear^6^; selective inactivation of the neurons in the CeA-LC reduces the acquisition of conditioned fear^4^; and inhibiting the relay of nociceptive information (the unconditioned stimulus) from neurons of the pontine parabrachial nucleus to CeA-LC neurons prevents fear-memory formation^7^. These findings show that excitatory and inhibitory synapses within the CeA-LC are important for the formation, consolidation and expression of fear memories^3^.

Neurons of the CeA receive excitatory connections from the basolateral amygdala (BLA), sensory cortices, the thalamus, and the pontine parabrachial nucleus (PBr)^3^. We have previously demonstrated that activation of presynaptic GABA_B_ receptors located on axon terminals of parabrachial neurons inhibits glutamate release onto to the CeA-LC^8^. However, the influence of GABA_B_ receptors in the CeA on synaptic transmission and fear memory formation has not been fully investigated. Here we demonstrate that presynaptic GABA_B_ receptors inhibit a number of excitatory and inhibitory connections within the CeA, and that activation of these GABA_B_ receptors, *in vivo*, during contextual fear conditioning blocks the formation of a context-shock fear memory.

## Results

**Figure 1:**
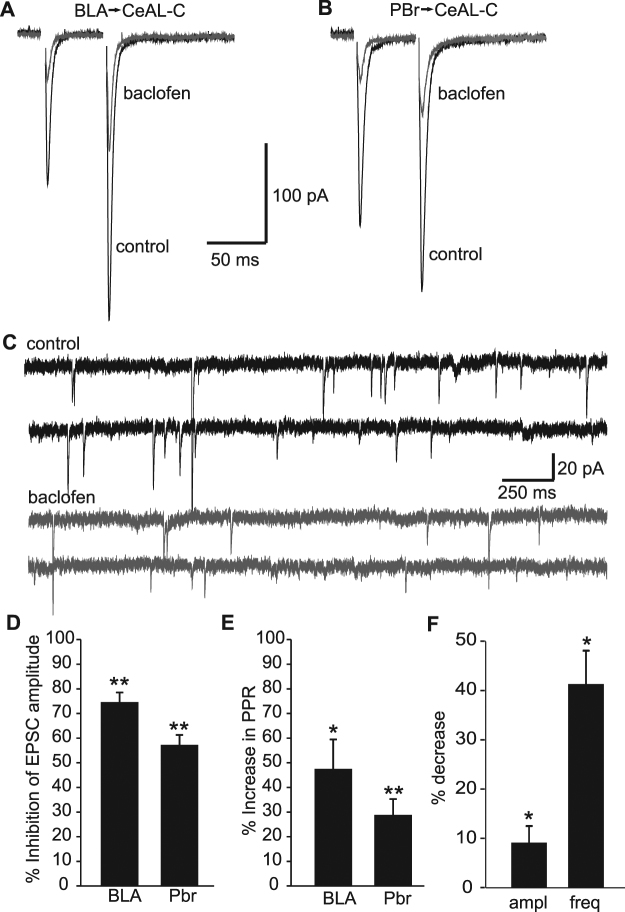
Effects of baclofen on excitatory currents in the CeAL-C. (**A**) Representative average BLA-evoked paired EPSCs recorded in control (black) and after application of baclofen (2 µM; grey). (**B**) Representative average PBr-evoked paired EPSCs recorded in control (black) and after application of baclofen (2 µM; grey). (**C**) Spontaneous EPSC currents recorded in control (black) and after addition of baclofen (2 µM; grey). Histograms showing the average percentage inhibition (±s.e.m) of evoked EPSC amplitude for BLA- and PBr-evoked EPSCs (**D**), the percentage increase (±s.e.m) in paired pulse ratio for BLA- and PBr-evoked paired EPSC (**E**) and the percentage decrease (±s.e.m) in amplitude and frequency for spontaneous EPSCs recorded in baclofen compared to control. (** denotes P < 0.01, * denotes P < 0.05).

We first sought to characterise the effect of the selective GABA_B_ agonist baclofen on excitatory synaptic transmission in the CeA-LC. Recording in voltage clamp mode with caesium-based internal solution, we electrically evoked excitatory post-synaptic currents (eEPSCs) at hyperpolarised membrane potential (−65 mV) in the presence of picrotoxin (100 µM) to block GABA_A_-mediated inhibitory currents. Stimulating in the lateral amygdala division of the BLA we found that baclofen (2 µM) reduced eEPSC amplitude by 74.7 ± 3.8% (Fig. 1A,D; p < 0.05, n = 6). Similarly, eEPSCs evoked by stimulating the ventral amygdalofugal axon bundle containing the axons from the PBr^8^ were inhibited by 57.3 ± 4.0% inhibition respectively (Fig. 1B,D; p < 0.05, n = 12 respectively). Inhibition of both eEPSCs was accompanied by a significant increase in paired pulse ratio (PPR), of 47.5 ± 11.9% and 28.9 ± 6.4% respectively (Fig. 1E; p < 0.05, n = 6 and 12 respectively), indicative of a reduction in pre-synaptic release probability. Furthermore, there was a significant decrease in sEPSC amplitude and frequency seen with the addition of baclofen (Fig. 1C,F; 9.0 ± 3.3 and 40.9 ± 6.7% reduction respectively, p < 0.05, n = 5), indicating a presynaptic change in vesicle release probability.

**Figure 2:**
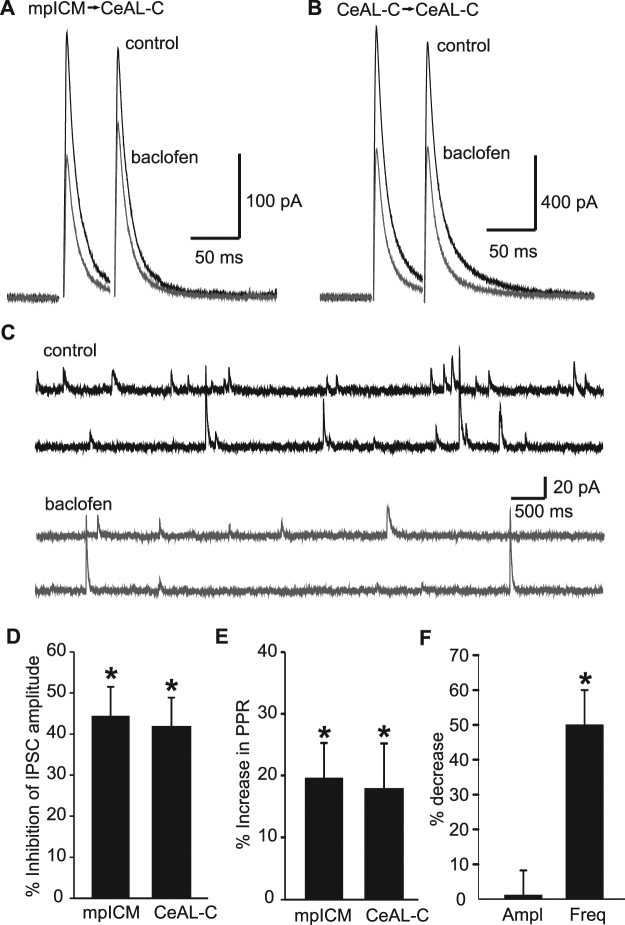
Effects of baclofen on inhibitory currents in the CeAL-C. (**A**) Representative average mpICM-evoked paired IPSCs recorded in control (black) and after application of baclofen (2 µM; grey). (**B**) Representative average CeAL-C-evoked paired IPSCs recorded in control (black) and after application of baclofen (2 µM; grey). (**C**) Spontaneous IPSC currents recorded in control (black) and after addition of baclofen (2 µM; grey). Histograms showing the average percentage inhibition (±s.e.m) of evoked IPSC amplitude for mpICM- and CeAL-C-evoked IPSCs (**D**), the percentage increase (±s.e.m) in paired pulse ratio for mpICM- and CeAL-C-evoked paired IPSC (**E**) and the percentage decrease (±s.e.m) in amplitude and frequency for spontaneous IPSCs recorded in baclofen compared to control. (* denotes P < 0.05).

Next, we characterised the effects of baclofen (2 µM) on inhibitory transmission in the CeA-LC. Electrically evoked monosynaptic inhibitory post-synaptic currents (eIPSCs) were recorded from CeA-LC cells using caesium based internal solution, at depolarised membrane potentials (−10 to 0 mV), and in the presence of 10 µM NBQX to block glutamatergic transmission. We stimulated electrically at the internal capsule fibre tract between the BLA and CeA-LC to activate inhibitory connections from the medial paracapsular intercalated cells (mpICM) from the anterior intercalated island^9^. Baclofen (2 µM) inhibited mpICM eIPSCs by an average 44.3 ± 7.1% (Fig. 2A,D; n = 5, p < 0.05). Inhibitory currents recorded in CeA-LC in response to local stimulation within the CeA-LC (medial to the recorded cell) were also sensitive to this concentration of baclofen (Fig. 2B; 42.6 ± 7.5% inhibition, n = 6, p < 0.05). Consistent with the actions of baclofen at the excitatory synapses just described, baclofen inhibition of the mpICM- and locally-evoked eIPSCs was also accompanied by a significant change in PPR (Fig. 2E; 19.8 ± 5.6% and 17.6 ± 7.2% increase, respectively, n = 5 and 6, p < 0.05). The frequency of spontaneous IPSCs recorded from CeA-LC neurons was also significantly decreased from an average frequency of 4.79 ± 0.78 Hz to 2.75 ± 1.04 Hz, (average 50.0 ± 10.0% reduction, n = 7, p < 0.01; Fig. 2F), however the amplitude of sIPSCs was unchanged (Fig. 2F). Together with the change in PPR of eIPSC, this result also suggests that baclofen acts presynaptically to reduce release at inhibitory terminals.

**Figure 3:**
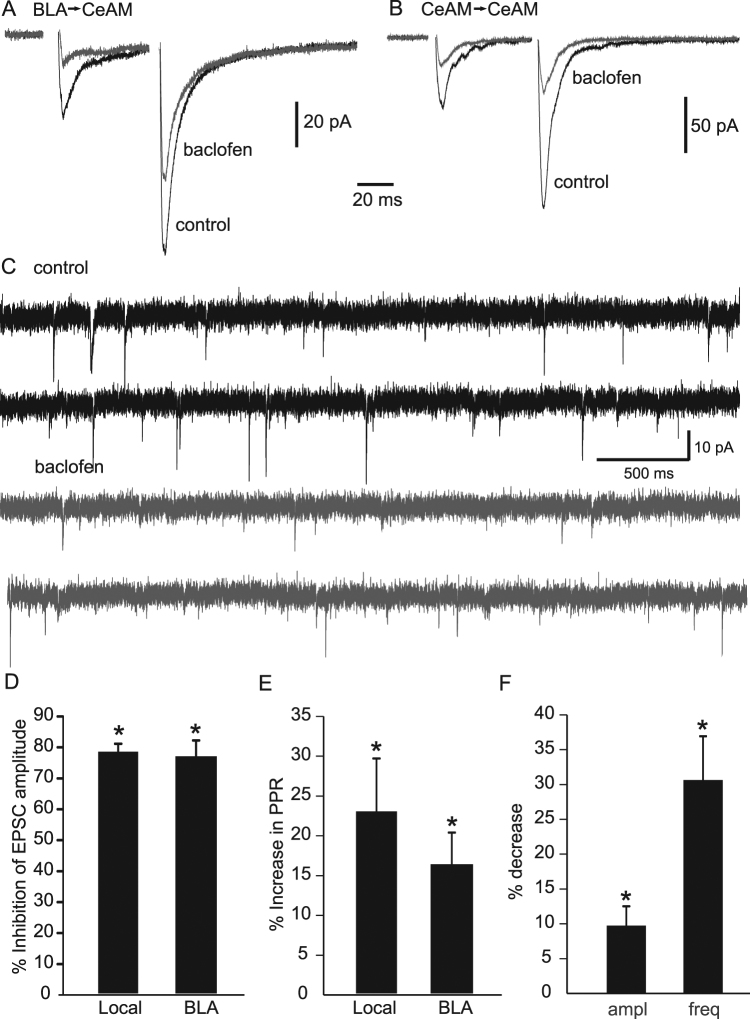
Effects of baclofen on excitatory currents in the CeAM. (**A**) Representative average BLA-evoked paired EPSCs recorded in control (black) and after application of baclofen (2 µM; grey). (**B**) Representative average local CeAM-evoked paired EPSCs recorded in control (black) and after application of baclofen (2 µM; grey). (**C**) Spontaneous EPSC currents recorded in control (black) and after addition of baclofen (2 µM; grey). Histograms showing the average percentage inhibition (±s.e.m) of evoked EPSC amplitude for BLA- and CeAM-evoked EPSCs (**D**), the percentage increase (±s.e.m) in paired pulse ratio for BLA- and CeAM-evoked paired EPSC (**E**) and the percentage decrease (±s.e.m) in amplitude and frequency for spontaneous EPSCs recorded in baclofen compared to control. (* denotes P < 0.05).

**Figure 4:**
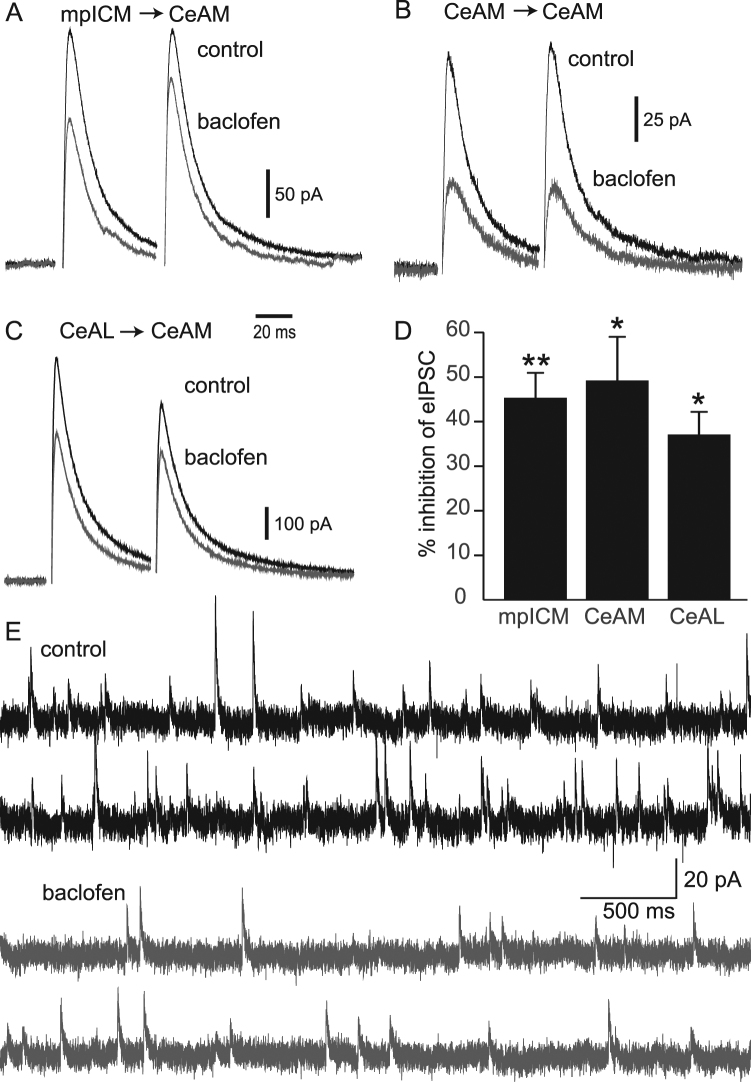
Effects of baclofen on inhibitory currents in the CeAM. (**A**) Representative average mpICM-evoked paired IPSCs recorded in control (black) and after application of baclofen (2 µM; grey). (**B**) Representative average CeAM-evoked paired IPSCs recorded in control (black) and after application of baclofen (2 µM; grey). (**C**) Representative average CeAL-C-evoked paired IPSCs recorded in control (black) and after application of baclofen (2 µM; grey). (**D**) Histograms showing the average percentage inhibition (±s.e.m) of evoked IPSC amplitude for mpICM-, CeAM- and CeAL-C-evoked IPSCs (* denotes P < 0.05). (**E**) Spontaneous IPSC currents recorded in control (black) and after addition of baclofen (2 µM; grey).

We then characterised the effects of baclofen on evoked excitatory and inhibitory currents recorded from CeA-M neurons. Baclofen significantly inhibited all excitatory and inhibitory activity. Excitatory responses in the CeA-M resulting from stimulation of the BLA were reduced by an average of 76.4 ± 3.8% (p < 0.05, n = 4, Fig. 3A,D) and eEPSC resulting from stimulation locally within the CeA-M were similarly reduced (Fig. 3B,D; 78.1 ± 2.5%, p < 0.05, n = 4). This reduction in eEPSC amplitude was accompanied by an increase in PPR for both BLA- and locally-evoked CeA-M (Fig. 3E, 16.1 ± 4.0% and 22.9 ± 6.7% respectively, p < 0.05, n = 4 for each). There was also a significant reduction in sEPSC amplitude and frequency with the addition of baclofen (9.7 ± 2.8% and 30.6 ± 6.3% respectively, p < 0.05, n = 5 for both). Inhibitory responses to stimulation of the internal capsule (mpICM), locally within the CeA-M, and within the CeA-LC, were all inhibited by baclofen (Fig. 4B–D; 45.4 ± 5.6%, 49 ± 9.8% and 37.1 ± 5.1%, inhibition respectively; p < 0.05 for each, n = 4, 4, and 7, respectively). This inhibition was associated with a significant increase in PPR for mpICM eIPSCs and CeA-LC eIPSCs (23.9 ± 9.5% and 11.6 ± 2.6% respectively, p < 0.05, n = 4 and 7, respectively), but no significant change in PPR was found for CeA-M eIPSCs (PPR change was 45.0 ± 29.8%, p = 0.28, n = 4). A reduction in spontaneous inhibitory amplitude and frequency was also seen with the application of baclofen (Fig. 4E,F; 15.3 ± 4.6% and 37.9 ± 8.1% inhibition respectively, p < 0.05, n = 12). Together, these results suggest that baclofen acts to reduce transmitter release at inhibitory and excitatory synapses in the CeA-M.

**Figure 5:**
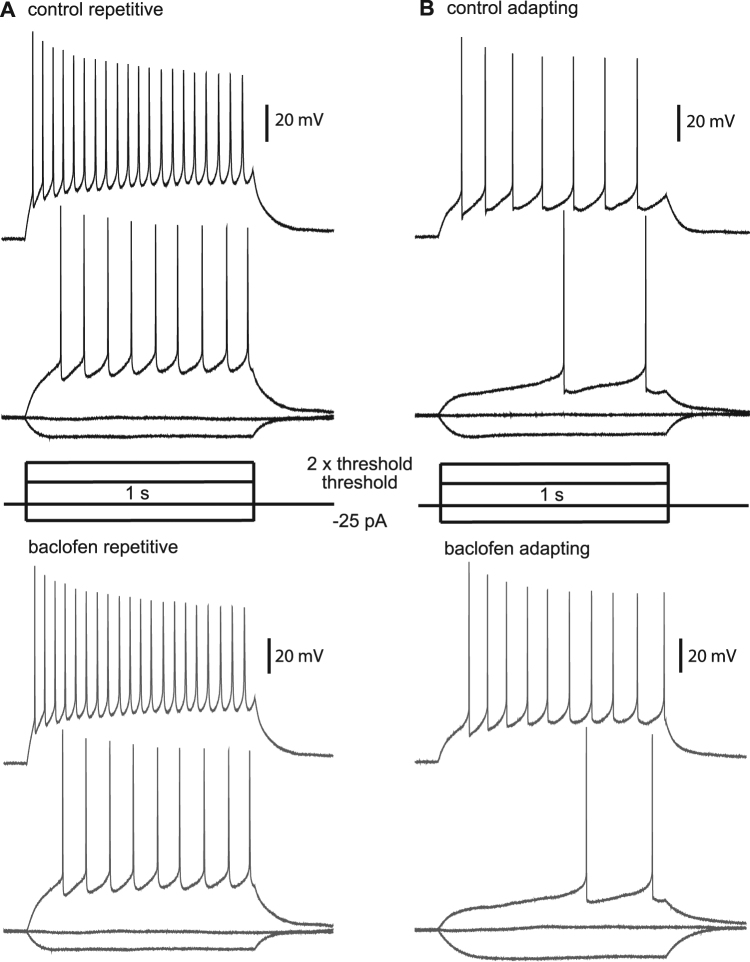
Effects of baclofen on action potential firing in CeAL-C neurons. (**A**) Firing pattern for a repetitive firing neuron in control conditions at 2x the threshold current injection (top) and at the threshold current injection (2^nd^ from top), compared to firing at 2x threshold (3^rd^ from top) and threshold (bottom) in the same neuron after application of baclofen. (**B**) Control and baclofen firing patterns for adapting type neurons in the same configuration as shown in A. Current injection steps are represented in the centre of A and B.

**Figure 6:**
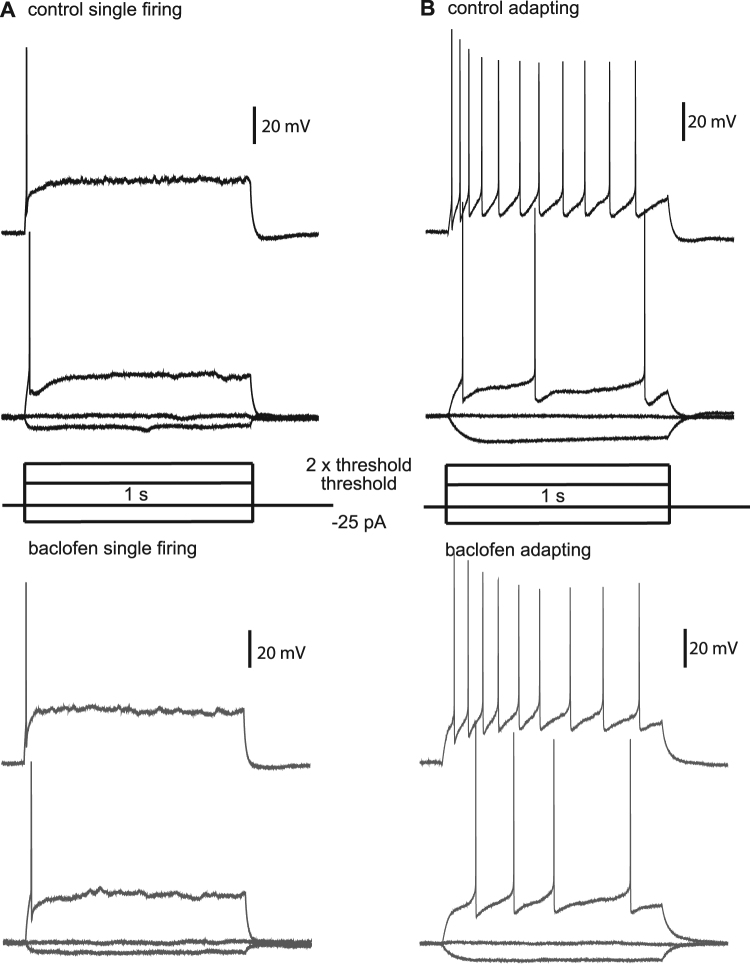
Effects of baclofen on action potential firing in CeAM neurons. (**A**) Firing pattern for a single firing neuron in control conditions at 2x the threshold current injection (top) and at the threshold current injection (2^nd^ from top), compared to firing at 2x threshold (3^rd^ from top) and threshold (bottom) in the same neuron after application of baclofen. (**B**) Control and baclofen firing patterns for adapting type neurons in the same configuration as shown in A. Current injection steps are represented in the centre of A and B.

Next, we examined the direct effects of baclofen on the cells in the CeA-LC and CeA-M. Two types of cells were distiguished in both the CeA-LC and CeA-M based on the firing pattern of these cells in response to increasing depolarising current injections (1 s in duration). In the CeA-LC, we grouped one type of cell where action potentials initially occur late in the current injection but display frequency adaptation with increasing depolarisation (adapting cell). The other type, classified as a repetitive firing cell, fired with uniform frequency during current injections, but the action potential firing frequency seen during each current injection increased as the size of the depolarisation increased. In the CeA-M we saw single firing neurons that produced only one or two action potentials with increased depolarisation and a type of adapting cells with firing similar to those seen in CeA-LC. These firing properties are consistent with those reported previously^10^. We measured the input resistance, action potential threshold and action potential width for the different cell types present in the CeA-LC and CeA-M in control and after application of baclofen. None of these were significantly changed by baclofen (Table 1), and no change in firing patterns for cells in the CeA-LC (Fig. 5) or the CeA-M (Fig. 6) was seen after the addition of baclofen.

**Table 1:** The effect of 2 µM baclofen (bac) on membrane and firing properties of CeAL-C and CeAM neurons.

Sub-division	Cell type	Number of cells	Resting membrane potential (mV)	Input resistance (MΩ)		Action potential threshold (mV)		Action potential width (ms)	
				*ctl*	*bac*	*ctl*	*bac*	*ctl*	*bac*
CeAL-C	Adapting	21	−63.4 ± 3.9	368 ± 194	418 ± 178	−35.1 ± 5.2	−35.5 ± 6.0	0.84 ± 0.11	0.84 ± 0.10
	Repetitive	7	−64.7 ± 5.5	386 ± 144	441 ± 205	−34.9 ± 4.1	−35.4 ± 6.1	0.87 ± 0.13	0.90 ± 0.13
CeAM	Single	6	−51.3 ± 5.5	216 ± 52	256 ± 51	−35.2 ± 5.5	−36.1 ± 4.0	0.72 ± 0.10	0.82 ± 0.12
	Adapting	11	−54.8 ± 5.1	409 ± 135	372 ± 120	−38.4 ± 4.0	−40.1 ± 3.8	0.74 ± 0.06	0.85 ± 0.27

**Figure 7:**
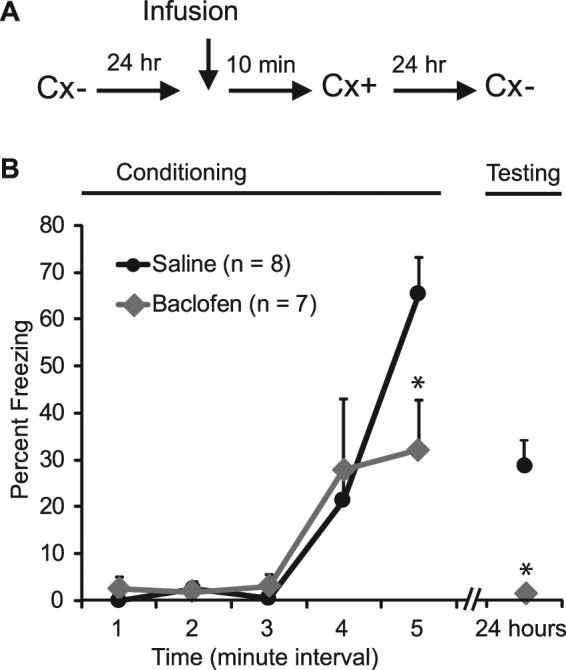
Baclofen infusions into the CeA block acquisition of context conditioned fear. (**A**) Schematic of the experimental design. The arrow indicates that baclofen infusions occurred 10 min prior to the onset of context-shock pairings (acquisition session). The graph shows the mean ± s.e.m. level of freezing in 1 min intervals during the acquisition session and in a 2 min test period 24 hours later for Groups Baclofen and Saline. (* denotes P < 0.05).

Finally, we tested the effect of baclofen mediated inhibition of synaptic inputs in the CeA on contextual fear-conditioning by infusing baclofen into the CeA (Fig. 7A). Rats were monitored for the duration of the 12 min infusion-context training period. While we made no objective measure of locomotion while the rats were in the conditioning chamber, we saw no obvious effects of the drug infusion on locomotor activity, posture or behaviors other than freezing while visually monitoring the rats during the conditioning or test sessions, or subsequently when performing off-line analysis of the session recordings. However, relative to vehicle-infused controls, we found that rats receiving a CeA infusion of baclofen immediately prior to context-shock pairings displayed less post-shock freezing across the conditioning session (Fig. 7B, p < 0.05). In addition, when these animals were exposed to the conditioned context in the absence of foot shock and in the absence of the drug 24 hours after training, those that had received baclofen-infusions during training again displayed significantly less freezing than animals that received vehicle-infusions (Fig. 7B, p < 0.05 for all time points, n = 7 baclofen, 8 vehicle).

## Discussion

The CeA complex plays a key role in the formation and expression of conditioned fear^3^. In the present study, we demonstrate that activation of presynaptic GABA_B_ receptors inhibits excitatory and inhibitory transmission in both divisions of the CeA complex. In addition, *in vivo* activation of GABA_B_ receptors in the CeA reduces the level of freezing during context fear conditioning (i.e., at the time when rats are exposed to context-shock pairings) and during a subsequent drug-free test. These results demonstrate that GABA_B_ receptors, by modulating synaptic transmission within the CeA complex, impair the formation and/or the expression of fear memories.

GABA_B_ receptors are G-protein-linked receptors that are activated by GABA, the primary inhibitory transmitter in the forebrain. In general, GABA_B_ receptors influence the activity of neurons in two ways: the activation of G-protein-gated inwardly rectifying channels (GIRKs) that reduce activity of postsynaptic neurons, and the inhibition of calcium channels within axon terminals that reduces activity-dependent neurotransmitter release^11^. Presynaptic GABA_B_ receptors modulate the activity of neural circuits in a number of brain regions^12–16^. Within the lateral amygdala, activation of presynaptic GABA_B_ receptors occurs as a result of the extra-synaptic spread of GABA, released by either local interneurons or GABAergic neurons of the basal division that project into the lateral amygdala^12,17^. The activation of GABA_B_ receptors located on glutamatergic terminals from the cortex and thalamus inhibits excitatory transmission onto pyramidal neurons of the lateral amygdala, preventing the development of long-term potentiation at these synapses^12^. Recently, we have demonstrated that activation of presynaptic GABA_B_ receptors, by inhibiting the activity of N-type calcium channels, reduces glutamate release from axons of PBr neurons in the CeA-LC^13^. In this study, we expand on this result by showing that activation of presynaptic GABA_B_ receptors inhibits a number of other excitatory and inhibitory connections within the CeA-LC and the CeA-M.

The majority of neurons in the CeA are GABAergic^18,19^. While GABAergic neurons in the CeA-LC are highly interconnected^5,6,20^, a subset (the CeA-LC_off_ neurons) also project onto neurons of the CeA-M^5^. In the lateral amygdala, repetitive stimulation of excitatory cortical inputs results in reduced glutamate release from excitatory thalamic inputs on to pyramidal cells, and similar inhibition of cortical inputs results from repetitive stimulation of thalamic inputs^12^. This heterosynaptic inhibition is due to cortical and thalamic excitation of local interneurons resulting in pooling of locally-released GABA and the subsequent activation of presynaptic GABA_B_ receptors located on the excitatory terminals^12^. Our results suggest that heterosynaptic inhibition could also reduce excitatory and inhibitory transmission in the CeA, influencing the formation and expression of fear memories. However, further studies are needed to determine whether pooling of locally-released GABA does indeed result in heterosynaptic inhibition within the CeA.

Neurons of the CeA-M initiate fear responses, and this action is under the control of neurons in the CeA-LC^4,5,21,22^. In addition, neurons in the CeA-LC are involved in the acquisition of fear memories^4,7^. Our results suggest that, by reducing transmission at synapses in the CeA, activation of GABA_B_ receptors influence fear memory formation and expression. Indeed, we found that infusions of the GABA_B_ receptor agonist baclofen into the CeA reduced both the freezing response seen at the end of the training phase and the freezing response to the conditioned context seen 24 hours after training. This result is consistent with the ability of presynaptic GABA_B_ receptors to inhibit almost all excitatory and inhibitory synapses that we tested within the CeA complex. For instance, the attenuated freezing response displayed in the final period of the training session could reflect the action of GABA_B_ receptors in two locations, the inhibition of excitatory drive onto CeA-M neurons and a decrease in excitatory drive onto CeA-LC neurons responsible for disinhibiting CeA-M neurons, both of which would reduce the expression of fear responses. Similarly, the reduced acquisition and/or consolidation of contextual fear memories (seen as a reduced freezing response 24 hours later) might involve GABA_B_ -mediated inhibition of excitatory inputs from the PBr onto the CeA-LC neurons that are needed for the acquisition of conditioned fear^7^. One limitation of the study is that we have only demonstrated these effects using a single dose of baclofen that likely produces a high level of GABA_B_ receptor activation. It would be of interest for future studies to determine whether these effects are maintained under lower doses of baclofen and how this might relate to the degree of GABA_B_ inhibition resulting from endogenous release of GABA within the CeA.

In conclusion, we have shown that activation of presynaptic GABA_B_ receptors inhibits a number of excitatory and inhibitory synapses within the CeA complex. Further, our results demonstrate that activation of these presynaptic GABA_B_ receptors can inhibit the acquisition of conditioned fear, providing a mechanism by which sustained activation of inhibitory neurons within the CeA complex could influence neural networks in this region. In addition, our results provide further evidence that neural networks within the CeA complex play a key role in the acquisition and expression of conditioned fear.

## Methods

### Electrophysiology

Acute brain slices were prepared from anaesthetized (isoflurane) 20–30 day old Sprague-Dawley rats. Rats were decapitated, and their brains quickly transferred into ice cold artificial cerebrospinal fluid (ACSF) containing 118 mM NaCl, 25 mM NaHCO_3_, 10 mM Glucose, 2.5 mM CaCl_2_, 1.2 mM NaH_2_PO_4_ and 1.3 mM MgCl_2_. The brains were then sliced into 350 µm thick coronal sections using a Leica VT1000S vibratome at 0 °C. The slices were then incubated in fresh ACSF for 30 mins at 33–34 °C. Brain slices were then incubated at room temperature prior to recording. All procedures were performed with the approval of the Institutional Animal Ethics Committees of Charles Sturt University, and carried out in accordance with the National Institute of Health *Guidelines for the Care and Use of Laboratory Animals*, revised 1996.

Individual brain slices were transferred into a recording chamber continuously perfused with oxygenated ACSF heated to 32–33 °C. Whole cell recordings were made from neurons visualized using IR/DIC techniques. Whole Cell voltage clamp recordings were made using 3–5 MΩ patch electrodes filled with internal solution comprising 135 mM CsMeSO_4_, 8 mM NaCl, 10 mM HEPES, 2 mM Mg_2_ATP and 0.3 mM Na_3_GTP (pH 7.2 with CsOH, osmolarity 290 mOsm/kg). Currents were amplified and filtered during recordings at 4–8 kHz by a Multiclamp 700A amplifier (Axon instruments, Foster City, CA), digitized at 20 kHz (National Instruments, USB-6221 digitiser), for recording by a Toshiba Satellite Pro L70 PC using Axograph software. During recording, access resistance was monitored and recordings were discontinued if access resistance changed by more than 10%. Access resistance was less than 15 MΩ for all recordings. Post-synaptic currents (PSCs) were stimulated electrically using bipolar stimulating electrodes. During recordings, picrotoxin, NBQX, and baclofen (Abcam) were applied to the slice via the perfusate.

10–50 individual stimulated responses were averaged to compare pre- and post drug responses. Paired Student’s T-tests were used for statistical comparisons between control and treatment (except where indicated). The results reported in the text are expressed as mean ± s.e.m and absolute percentage change for changes from control.

### Contextual Fear conditioning

#### Subjects

Experimentally naïve, male Sprague-Dawley rats (320–400 g; Animal Resources Centre, Perth, Western Australia) were housed in groups of four in conventional cages (67 cm length × 40 cm width × 22 cm height) located in a climate controlled room maintained on a 12:12 light/dark cycle. Rats had free access to standard chow and water. All rats were handled each day (3 mins) for seven days prior to the commencement of experiments. All experimental procedures were approved by the Animal Care and Ethics Committee at the University of New South Wales and in accordance with the National Institute of Health *Guidelines for the Care and Use of Laboratory Animals*, revised 1996. 8 rats were randomly assigned to either the Drug (baclofen) group, or Vehicle (saline) group.

#### Behavioural Apparatus

Conditioning chambers were constructed from clear Perspex (front and back wall) and aluminium (sidewalls and ceiling) and measured 30 cm long, 27 cm wide and 30 cm high. The chamber floor was made of 2 mm diameter stainless steel rods spaced 13 mm apart. During experiments, a custom-build constant-current shock generator delivered a 0.5 s duration unscrambled alternating current (AC) 50 Hz shock at 0.8 mA intensity to the floor of each chamber. A tray below this floor was filled with bedding material (corncob). During use, chambers were placed in a sound- and light-attenuating cabinet with infrared illumination (940 ± 25 nm). A camera (Sony CCD 420TVL) was mounted to record the behaviour of each rat during training and test sessions through the back wall of the conditioning chamber. The background noise level was monitored using a digital sound-level meter (Dick Smith Electronics, Australia) and was less than 50 dB during experiments. Live camera images were monitored and recorded to DVD in an adjacent room. Experimental events (i.e., foot-shock) were computer generated using MATLAB software, (MathWorks Inc.). All chambers were cleaned using water following use.

#### Surgery

Rats were anaesthetized with co-administration of ketamine (Ketapex; Apex Laboratories, Sydney, Australia) and the muscle relaxant, xylazine (Rompun; Bayer, Sydney, Australia), Ketamine (100 mg/ml) was administered at a dose of 1.0 ml/kg (i.p.), and xylazine (20 mg/ml) was administered at 0.3 ml/kg. Once anesthetized, rats were place in stereotaxic apparatus (Kopf Instruments, Tujunga, CA), for intracranial surgery. Guide cannulas (26 gauge, 11 mm in length, Plastics One, Roanoke, VA) targeting the CeA of both hemispheres were implanted bilaterally. The following coordinates were used to target the CeA: 2.3 mm posterior to bregma, 4.2 mm lateral to the midline, and 8.0 mm ventral to the skull^23^. Implanted guide cannulas were secured with dental cement and four jeweller’s screws to the skull. Dummy cannulae extending 1 mm beyond the end of the guide cannulae, were kept in each guide at all times other than when they were removed for microinjections. Rats were administered a prophylactic (0.3 ml) dose of 300 mg/kg solution of procaine penicillin following surgery (ip injection) and allowed seven days to recover from the surgery. During recovery, handling continued (3 mins per day), and rats were monitored for changes in weight and behaviour.

#### Drug Infusions

A total volume of 0.2 μL, of 6.25 μg/μL Baclofen (Sigma, Australia) dissolved in non-pyrogenic saline, was infused into the CeA in each hemisphere via internal infusion cannulae (33-gauge) inserted into the guide cannulae. The concentrations and volumes were selected on the basis of our previous study in which we infused clonidine, which has an identical molecular weight to baclofen, and observed localized effects of the infusion on context fear conditioning^24^. The infusions occurred over one minute and the infusion cannulas remained in place to allow for diffusion of the drug or vehicle for two minutes post-infusion. The time between the removal of the infusion cannulae and the onset of training in the experimental chamber was exactly seven minutes.

#### Context conditioning

Day 1: Rats were pre-exposed to the experimental chambers for five minutes to reduce neophobia and to increase context conditioned fear^25,26^.

Day 2: Rats received bilateral CeA infusions of either baclofen or saline (as described above) before they were placed in the experimental chambers for context-shock pairings for acquisition. Rats received two shocks: one delivered three min after placement into the chamber, and the second at four min. Rats then remained in the chamber for an additional minute and the total time for acquisition was five minutes.

Day 3: Animals were returned to the experimental chambers (drug free) for testing. They were placed in the context for 10 minutes in the absence of foot shock.

#### Data Collection and Analysis

Freezing was used to assess fear during acquisition and testing. Following convention, we defined freezing as the absence of all movement except that required for breathing^25^. During off-line analysis of DVD recordings, the freezing behaviour for each rat was measured by an observer who was blind to the experimental conditions and group allocations. Observations made in 2 second intervals were scored as either ‘*freezing’* or ‘*not freezing*’. A percentage freezing score was then calculated by dividing the number of samples scored as freezing by the total number of samples scored for each animal (converted to a percentage). Data was compiled in Microsoft Excel (Microsoft Office, 2011) and analysed using a mixed model Analysis of Variance with a between-subject factor of treatment condition (Drug vs. Saline) and a within-subject factor of session time (blocks of 2 min). To maintain the chance of a Type 1 error at alpha = 0.05, the criterion for rejection of the null hypothesis (F_Critical_) was set at F_1,10_ = 4.96.

#### Histology

Immediately after testing, rats were administered a lethal dose of sodium pentobarbital (i.p.), decapitated, and their brains were removed and frozen. The frozen brains were subsequently serially sectioned through the CeA (coronal sections at 40 µm), and every second section collected on a glass slide and stained with cresyl violet. The locations of the implanted canula tips were then determined under a microscope using the boundaries of the CeA defined by Paxinos and Watson^23^. The location of the guide cannula tips for rats infused with baclofen or vehicle are provided in Supplementary Fig. 1. The plotted points in these figures represent the ventral point of the cannula track. One rat in the drug group had misplaced cannulas and was excluded from the statistical analysis. The final group sizes were baclofen, *n* = 7, and saline, *n* = 8.

### Data availability

The datasets generated during and/or analysed during the current study are available from the corresponding author on reasonable request.

## Electronic supplementary material

Supplementary Information
