# Supplementary material for: Baclofen acts in the central amygdala to reduce synaptic transmission and impair context fear conditioning
Source: Sci Rep. 2018 Jul 2;8:9908. doi: 10.1038/s41598-018-28321-0 (PMC6028433; doi:10.1038/s41598-018-28321-0)
Supplement: Supplementary file 1 — Supplementary Information [file 41598_2018_28321_MOESM1_ESM.docx]

**Baclofen blocks excitatory and inhibitory transmission in the central amygdala and reduces learning in context conditioning.**

**A.J. Delaney^1*^, J.W. Crane^1^, N.M. Holmes^2^, J. Fam^2^, and R.F. Westbrook^2^**

**Author Affiliation**

1. School of Biomedical Sciences, Charles Sturt University, Orange NSW, Australia 2800
2. School of Psychology, University of New South Wales, Sydney NSW, Australia 2052.

*Corresponding Author

Andrew J. Delaney

Senior Lecturer in Anatomy and Physiology

School of Biomedical Sciences,

Charles Sturt University,

Leeds Parade, Orange, NSW,

Australia, 2800

Email – [adelaney@csu.edu.au](mailto:adelaney@csu.edu.au)

**Supplementary Information**


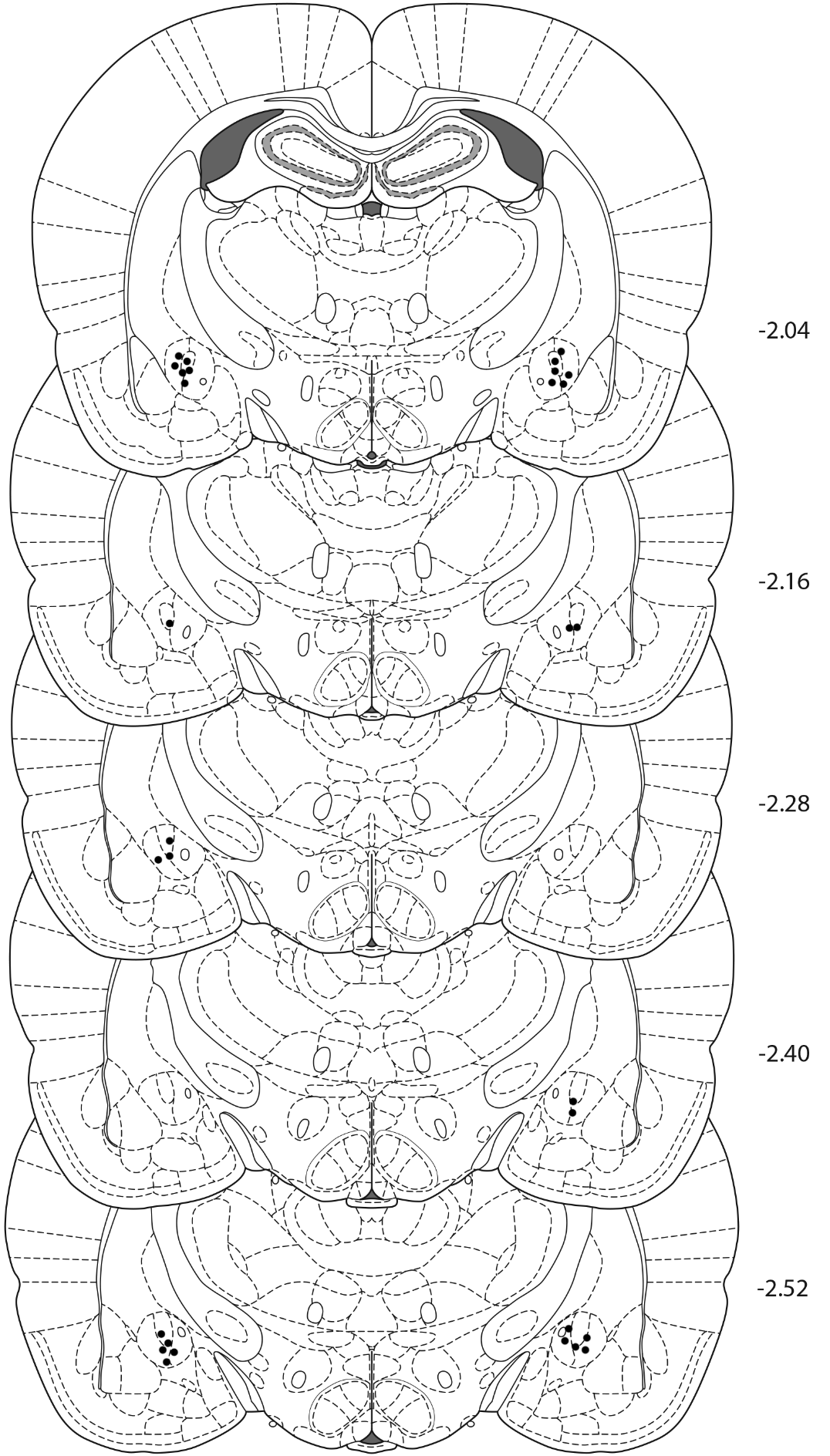


Supplementary Figure 1: Location of cannula placements for baclofen and vehicle infusions into the CeA for context conditioning experiment 1. Only subjects with bilateral cannula placements in the CeA are represented.
